# Efficacy and Safety of Percutaneous ASD Closure in Adults: Comparative Outcomes of Occluder Devices in a Single-Center Cohort

**DOI:** 10.3390/jcm14061867

**Published:** 2025-03-10

**Authors:** Elham Kayvanpour, Elena Matzeit, Christoph Reich, Ziya Kaya, Sven Pleger, Norbert Frey, Benjamin Meder, Farbod Sedaghat-Hamedani

**Affiliations:** 1 Department of Internal Medicine III, Heidelberg University, 69120 Heidelberg, Germany; elham.kayvanpour@med.uni-heidelberg.de (E.K.);; 2 DZHK (German Center for Cardiovascular Research), Partner Site Heidelberg/Mannheim, 69120 Heidelberg, Germany

**Keywords:** ASD closure, congenital heart disease, new-onset atrial fibrillation

## Abstract

**Background:** Atrial septal defect (ASD) is a prevalent congenital heart condition, resulting in left-to-right shunting. Untreated ASDs may be associated with complications, including right-sided heart failure, pulmonary hypertension, and atrial arrhythmias. Percutaneous ASD closure, performed with various occluder devices, has become the preferred approach for symptomatic patients with suitable anatomy, yet data on device-specific efficacy and safety profiles remain limited. **Methods:** This study was a retrospective, single-center analysis involving patients who underwent percutaneous ASD closure between January 2000 and February 2023. Data on patient characteristics, indications for the procedure, procedural details, and clinical outcomes were extracted from electronic medical records. Endpoints included complications at the puncture site, pericardial effusion, atrial arrhythmias, device-related thrombus formation, and overall survival. **Results:** A total of 195 patients were included (mean age 53.6 ± 16.2 years; 60.5% female). Three different devices were used: Amplatzer ASD occluder (*n* = 111), Gore Septal Occluder (*n* = 67), and Occlutech ASD occluder (*n* = 17). Initial procedural success rate was 90.8%, with no significant differences observed between devices. Periprocedural complication rates were low and comparable across all devices. New-onset atrial fibrillation within the first month post-implantation occurred in 7.5% of patients with the Gore device, compared to 0.9% with the Amplatzer device (*p* = 0.03) and 0% with the Occlutech device. No statistically significant differences were observed among the devices regarding thrombus formation, late-onset pericardial effusion, device erosion, or stroke. **Conclusions:** Percutaneous ASD closure demonstrates high procedural success and low complication rates across different occluder devices, supporting its efficacy and safety as a treatment for adults. Although the Gore device showed a higher incidence of new-onset AF compared to the Amplatzer device, no significant differences were observed regarding thrombus formation, pericardial effusion, device erosion or stroke.

## 1. Introduction

Atrial septal defect (ASD) is among the most common congenital cardiac anomalies, characterized by an abnormal septal opening that enables oxygenated blood from the left atrium (LA) to flow into the right atrium (RA). This left-to-right shunt increases blood volume in the right heart and pulmonary circulation, predisposing patients to a range of serious complications if left untreated [1]. Over time, unrepaired ASDs can lead to right ventricular (RV) volume overload, pulmonary hypertension, atrial arrhythmias, and heart failure. Although small ASDs may remain asymptomatic throughout life, larger defects are typically associated with symptoms in adulthood, including exertional dyspnea, fatigue, and palpitations [2,3]. Historically, surgical closure of ASDs was the primary treatment. However, in recent years, percutaneous ASD closure has become the preferred treatment approach due to its minimally invasive nature, expedited recovery, and reduced complication rates compared to surgical repair [4].

To accommodate anatomical variations in ASD morphology, several specialized occluder devices have been developed and refined [5]. Notable devices in current use include the Occlutech ASD Occluder (Occlutech GmbH, Jena, Germany), Amplatzer ASD Occluder (Abbott Laboratories, Chicago, IL, USA), and Gore Helex Septal Occluder (W. L. Gore & Associates, Inc., Flagstaff, AZ, USA). Each device offers distinct design features to optimize procedural success, but while multiple studies have validated their general safety and efficacy, data comparing device-specific outcomes—such as rates of residual shunt, device thrombus formation, and arrhythmias—are limited. The need for device-specific safety and efficacy data in diverse clinical settings remains essential to refine device selection and minimize risks.

**Figure 1:**
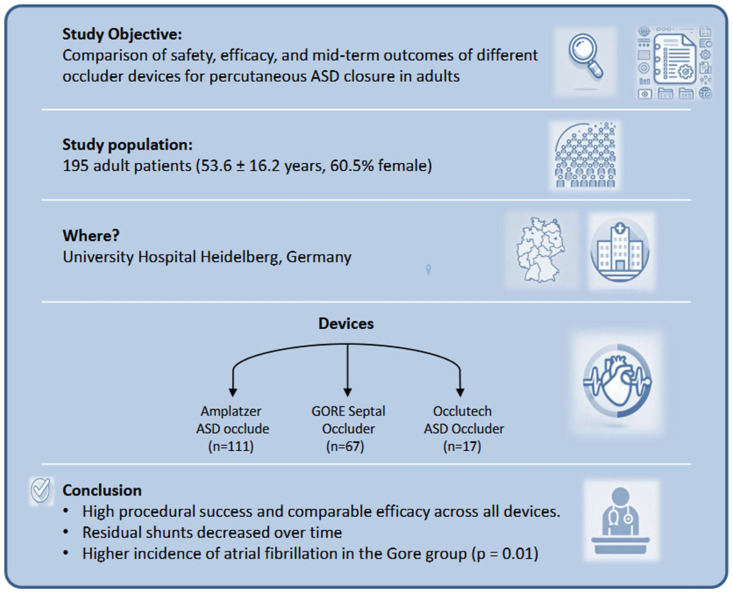
Summary of the study comparing percutaneous ASD closure outcomes across different occluder devices. The graphical abstract highlights the study population, procedural success, residual shunt rates, and device-related complications, emphasizing the need for individualized device selection.

In this study, we retrospectively analyzed the outcomes of percutaneous ASD closure in a cohort of adult patients treated at our center. The primary objective was to compare the performance of different occluder devices in terms of procedural success, safety, and mid-term outcomes. Specifically, we assessed the incidence of residual shunts, device-related complications, and changes in pulmonary artery systolic pressure over a 12-month follow-up period (Figure 1).

## 2. Materials and Methods

### 2.1. Study Design and Population

This retrospective, single-center observational study was conducted at University Hospital Heidelberg and focused on adult patients undergoing percutaneous ASD closure. The cohort included 195 patients treated between January 2000 and February 2023. Inclusion criteria were age ≥18 years at the time of procedure and a confirmed diagnosis of ASD with significant left-to-right shunting requiring closure. The study protocol adhered to ethical guidelines and was approved by the institutional review board (reference number S-060/2023), with a waiver of informed consent due to its retrospective design.

### 2.2. Data Collection

Data were extracted from electronic medical records, providing a comprehensive set of demographics, clinical, and procedural variables. Collected variables included patient age, sex, body mass index (BMI), underlying cardiovascular conditions, and relevant echocardiographic parameters. Information on ASD closure indications, procedural details, and outcomes was documented. Follow-up data were collected at 1, 6, and 12 months post-procedure to assess closure success and any complications. All data were anonymized prior to analysis.

### 2.3. ASD Closure Procedure and Device Selection

Percutaneous ASD closures were performed by experienced interventional cardiologists using standardized techniques under conscious sedation or general anesthesia, based on patient status. Device selection was guided by anatomical considerations and physician expertise, with devices used in this study including the Occlutech ASD Occluder (Occlutech GmbH, Jena, Germany), Amplatzer ASD Occluder (Abbott Laboratories, Chicago, IL, USA), and Gore Helex Septal Occluder (W. L. Gore & Associates, Inc., Flagstaff, AZ, USA). All procedures utilized femoral vein access, with device deployment guided by transesophageal echocardiography (TEE) to ensure optimal positioning. Post-procedural care involved echocardiographic assessments to confirm closure success and detect any complications.

### 2.4. Outcome Measures

The main outcomes were procedural success rates and residual shunt incidence at 1 and 6 months. Further outcomes included post-procedural complications such as device-related thrombus, pericardial effusion, vascular access complications, and new-onset atrial fibrillation or flutter. Additional events, including stroke, transient ischemic attack (TIA), and mortality, were also recorded during the follow-up period. Outcome assessments were based on clinical exams and echocardiographic evaluations at follow-up.

### 2.5. Statistical Analysis

Baseline characteristics, procedural details, and outcomes were summarized using descriptive statistics. Continuous variables were presented as means with standard deviations or medians with interquartile ranges as appropriate, while categorical variables were expressed as counts and percentages. Device comparisons utilized chi-square or Fisher’s exact tests for categorical variables and ANOVA or Kruskal–Wallis tests for continuous variables, with statistical significance defined as *p* < 0.05. All statistical analyses were performed using R (version 4.2.0; R Foundation for Statistical Computing, Vienna, Austria) and SPSS Statistics (version 21; IBM Corp., Armonk, NY, USA).

## 3. Results

### 3.1. Patient Characteristics and Demographics

A total of 195 patients who underwent percutaneous ASD closure were included in the study. The mean age of the cohort was 53.6 ± 16.2 years, with 60.5% being female (Table 1). Clinical parameters, including rates of atrial fibrillation/flutter, arterial hypertension, smoking, diabetes mellitus, history of syncope, and pulmonary hypertension were generally comparable among the groups, with no statistically significant differences observed. Hemodynamic measurements, such as systolic pulmonary pressure (PaSys) and shunt size as a percentage of cardiac output, showed slight variation but did not reach statistical significance.

The most frequent indications for ASD closure were hemodynamic relevant shunt (35.9%), stroke or transient ischemic attack (TIA) (34.9%), and right heart overload (15.9%) (Table 2).

### 3.2. Procedural Data and Periprocedural Complications

Procedural success was achieved in 90.8% (177/195) of cases, with no statistically significant differences in success rates across devices (*p* = 0.6). The Gore Septal Occluder had a significantly shorter average procedure duration (46.5 ± 25.4 min) compared to the Amplatzer ASD occluder (55.3 ± 23.8 min) and Occlutech ASD occluder devices (48.8 ± 23.6 min) (*p* = 0.04). Additionally, fluoroscopy time was significantly shorter for the Gore Septal Occluder, averaging 7.0 ± 6.0 min, compared to 9.7 ± 6.8 min for the Amplatzer ASD occluder and 8.4 ± 5.3 min for the Occlutech ASD occluder (*p* = 0.02) (Table 3). Periprocedural device dislocation was observed in one case during Amplatzer ASD occluder implantation, and a puncture site complication with an arteriovenous (AV) fistula occurred in one case during Gore Septal Occluder implantation. No cases of hemodynamically significant pericardial effusion, TIA/stroke, bleeding, or death were reported peri-procedurally (Supplementary Table S1). Manual compression was generally used for venous access site hemostasis, with pressure applied for approximately 5–10 min, followed by the application of a pressure bandage if no further bleeding was observed.

A case of device dislocation occurred in a 27-year-old patient with a secundum ASD (10 × 13 mm, Qp/Qs = 2.2) associated with an atrial septal aneurysm and a Chiari network. An Amplatzer ASD Occluder (15 mm) was selected, but multiple deployment attempts resulted in misalignment. During the final positioning, the occluder initially appeared well-positioned; however, after release, the device embolized and was successfully retrieved via femoral artery access.

### 3.3. Trends in NYHA Class Transitions

**Figure 2:**
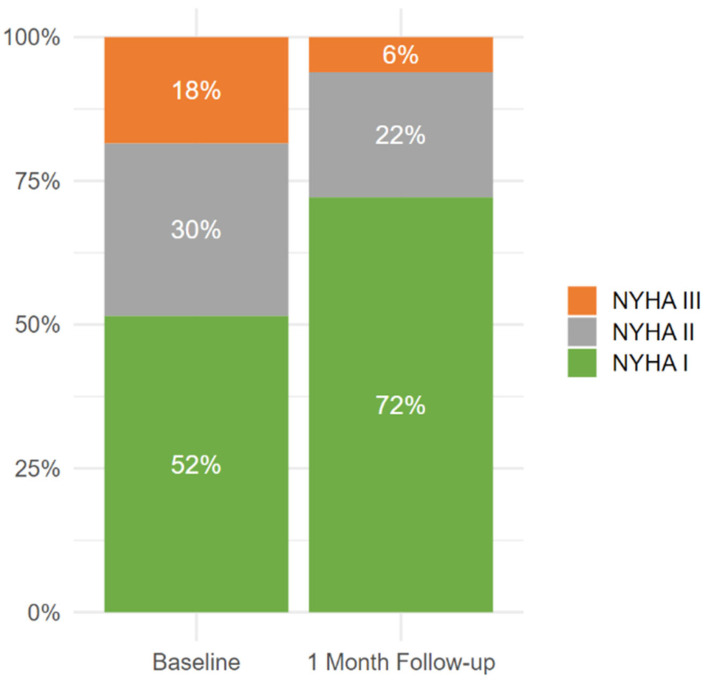
Changes in NYHA classification between baseline and one month after ASD occlusion.

At baseline, 51.5% of patients were in NYHA class I, increasing to 72.2% at the 1-month follow-up, indicating a significant improvement in functional status. Concurrently, the proportion of patients in NYHA class III decreased notably from 18.5% to 6.1%, and those in NYHA class II showed a reduction from 30% to 21.7%. No patients were classified as NYHA IV at either time point. These findings demonstrate a clear trend toward improved functional status within one month of ASD closure, with a substantial shift of patients from higher NYHA classes (II and III) to lower classes (I and II), highlighting the procedure’s early clinical benefits (Figure 2).

### 3.4. Residual Shunt

TEE is routinely performed after implantation to assess device positioning, detect residual shunt (including bubble test), and evaluate the presence of thrombus formation. One-month residual shunt was observed in 22.7% of patients. Residual shunt rates were 20% in the Occlutech group, 18.8% in the Amplatzer group, and 29.1% in the Gore group, with no statistically significant differences among devices (*p* = 0.5). By 6 months, residual shunt rates further decreased to 13.5%. All residual shunts observed were peri-prosthetic, with no cases of intra-prosthetic shunting. Consequently, the self-centering or non-self-centering design of the occluders did not appear to influence the sealing mechanism in our cohort.

### 3.5. Post-Procedural Events

Device-specific analysis indicated a significantly higher incidence of new-onset atrial fibrillation already during the first month after ASD occlusion in the Gore device group, with 7.5% of patients experiencing new-onset atrial fibrillation, compared to 0.9% in the Amplatzer group (*p* = 0.03) and none in the Occlutech group. This finding suggests a potential device-specific association with atrial arrhythmias, particularly with the Gore device.

During the one-year follow-up, device-related thrombus formation was observed in 1% (*n* = 2) of patients, exclusively in the Gore group. Stroke or TIA was rare across all device groups, occurring in only 1% (*n* = 2) of cases, underscoring the low thromboembolic risk associated with percutaneous ASD closure. Notably, no cases of late-onset pericardial effusion leading to tamponade were observed, highlighting the safety of this procedure (Table 4).

The first stroke occurred in a 63-year-old patient with chronic atrial fibrillation who developed a cerebellar infarction 12 days after ASD closure with a 30 mm Gore Septal Occluder, despite being on apixaban therapy and showing no thrombus on TEE. The second event involved a 33-year-old patient with Hodgkin’s lymphoma and a prior stroke, who experienced recurrent neurological symptoms three months after closure with a 25 mm Gore Septal Occluder. TEE showed no thrombus, but a pulmonary shunt suggested paradoxical embolization due to tumor-associated thrombophilia. The patient was subsequently switched from aspirin to phenprocoumon, with no atrial fibrillation detected.

Two cases of device-related complications due to dislocation or embolization, requiring surgical intervention, were observed during follow-up. The first case involved a 44-year-old patient with a large ASD II (13 × 20 mm) and suspected paradoxical coronary embolism who underwent percutaneous ASD closure. However, four weeks post-procedure, TEE revealed embolization of the device into the aortic arch. An interventional retrieval attempt via catheterization was unsuccessful, necessitating surgical ASD closure and device removal. The second case involved a 47-year-old patient with a hemodynamically relevant ASD II (16 × 14 mm) and a history of TIA, in whom follow-up TEE showed device dislocation with tilting at the aortic rim and significant residual shunting. Given these findings, surgical explantation and ASD closure were successfully performed.

One case of death occurred approximately two months after ASD closure in a patient with pre-existing dilated cardiomyopathy (DCM), entirely unrelated to the procedure or the device. The patient developed a COVID-19 infection with pneumonia, which led to cardiogenic shock due to pre-existing heart failure and severely impaired left ventricular function. The cause of death was a combination of pulmonary and cardiovascular factors, independent of the ASD closure.

### 3.6. Pulmonary Artery Pressure Changes

**Figure 3:**
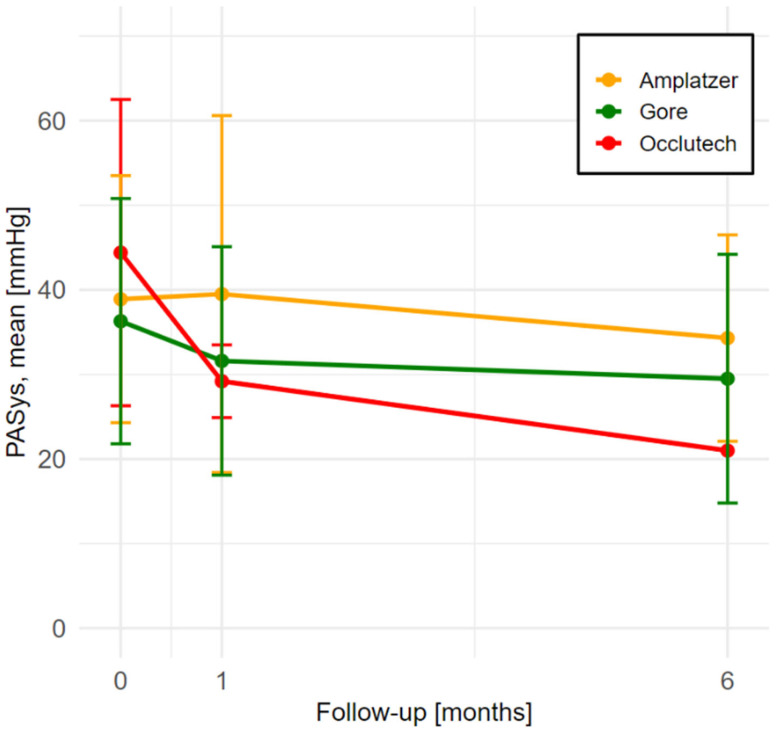
Changes in systolic pulmonary artery pressure (PaSys) values across four time points, categorized by ASD occluder device.

Figure 3 summarizes the PaSys values across four time points for patients treated with three different ASD occluder devices. At baseline, no significant differences were observed among the groups (*p* = 0.3), with values of 44.4 ± 18.1 mmHg in the Occlutech group, 38.9 ± 14.6 mmHg in the Amplatzer group, and 36.3 ± 14.5 mmHg in the Gore group. At the 1-month follow-up, a significant difference emerged (*p* = 0.02), as the Occlutech group demonstrated the most pronounced reduction in PaSys compared to the Amplatzer and Gore groups. By the 6- and 12-month follow-ups, PaSys declined further across all groups. These findings highlight the beneficial outcomes of percutaneous ASD closure in reducing pulmonary pressure and improving overall cardiac function, regardless of the device used.

## 4. Discussion

This study presents a comparative analysis of percutaneous ASD closure in adults using three commonly utilized occluder devices: Occlutech, Amplatzer, and Gore septal occluder. The findings highlight the safety and efficacy of ASD closure, with high procedural success rates across all devices and a low incidence of major complications, consistent with previous reports on ASD closure outcomes [5,6,7].

The observed improvement in NYHA functional status within one month of ASD closure underscores the rapid symptomatic relief provided by the procedure. The significant rise in NYHA class I suggests a rapid recovery of functional capacity, particularly among patients with less severe symptoms at baseline. The reduction in NYHA class III, alongside the stabilization of NYHA class II proportions, reflects a meaningful improvement even in moderately symptomatic patients. The absence of patients in NYHA class IV further underscores the timely intervention in this cohort, potentially preventing more severe symptom progression. These findings align with existing evidence supporting ASD closure as an effective strategy for improving functional status in the short term, with potential implications for long-term cardiovascular health and patient outcomes [8].

The Gore device exhibited significantly shorter procedural and fluoroscopy times. This efficiency may be attributed to the unique design of the Gore device, which integrates the delivery sheath with the occluder, facilitating a more streamlined deployment process. However, a notable finding was the significantly higher incidence of new-onset AF in patients with the Gore device. This difference raises important considerations about device structure and its impact on atrial mechanics. Larger device profiles and local atrial irritation could trigger an inflammatory response triggering arrhythmia such as AF [9,10,11]. The Gore device’s unique composition, designed for flexibility and rapid endothelialization, may paradoxically contribute to increased atrial irritation to structural interactions with the septal tissue. In line with our findings, previous studies report no significant differences in new-onset AF between the Occlutech and Amplatzer occluders following ASD closure [12,13]. These results underscore the importance of selecting devices that not only meet anatomical requirements but also account for each patient’s specific risk factors for arrhythmias. For patients with pre-existing atrial arrhythmias or other risk factors for AF, careful consideration should be given to the potential for increased atrial strain with specific devices, and rhythm monitoring may be particularly warranted in the post-procedural period for those receiving the Gore device.

Residual shunting after ASD closure is a well-documented phenomenon [14]. Small peri-prosthetic or intra-prosthetic shunts typically resolve over time, likely due to endothelialization and progressive device integration. In contrast, larger peri-prosthetic shunts tend to persist and are often associated with anatomical variations, suboptimal rim engagement, or incomplete device apposition. The progressive reduction of residual shunts observed in our study aligns with previous reports demonstrating spontaneous closure within the first year post-procedure [5,12]. In our cohort, all persistent residual shunts were peri-prosthetic, with no cases of intra-prosthetic shunting at follow-up. The comparable residual shunt rates across all three devices suggest that the self-centering or non-self-centering design did not significantly influence the sealing mechanism. These findings emphasize that the primary determinant of persistent shunting may be anatomical factors rather than device structure alone. However, a recent systematic review and network meta-analysis indicated a higher incidence of residual shunting with the Gore occluder compared to the Amplatzer device, potentially due to structural differences affecting shunt resolution [5]. This highlights the need for further investigations to determine whether specific device characteristics contribute to prolonged peri-prosthetic shunting. A prospective, randomized study could provide further insights into device-specific factors influencing residual shunt rates and help refine optimal device selection to improve long-term clinical outcomes.

The risk of device erosion is a critical complication in ASD closure procedures. Erosion incidents are often linked to anatomical factors like deficient aortic rims and oversizing, where repetitive contact between the device and the aortic or atrial wall leads to tissue injury [15,16]. This complication typically arises within the first six months post-implantation, though cases have been documented as late as nine years after the procedure [6]. In our study, no cardiac erosion was detected during the 12-month follow-up; however, prior data report an erosion rate of 0.1 to 0.3% associated with the Amplatzer occlude [15,17]. By contrast, the Occlutech and Gore occluders, with their more flexible structures and adaptable designs, may offer advantages for patients with limited aortic rim support, such as those with short or deficient rims [6,12,15,16]. This difference in erosion risk highlights the importance of careful patient selection and precise device sizing to minimize the risk of erosion and enhance patient safety [15].

Our study observed significant reductions in PAsys within one month of ASD closure in the Occlutech group, with sustained reductions through the 12-month follow-up across all three devices. This aligns with findings from other studies that report significant PAsys reduction post-closure, likely due to decreased left-to-right shunting and subsequent alleviation of pulmonary vascular load. The consistent PAsys reductions across all device groups highlight that the hemodynamic benefits of ASD closure encompass all device types, making percutaneous closure a beneficial intervention for patients regardless of device selection. Additionally, previous studies have demonstrated that ASD closure in elderly patients leads to significant functional improvement, enhanced quality of life, and favorable cardiac remodeling [18,19]. The observed benefits extend beyond younger populations, emphasizing the procedure’s positive impact on long-term cardiovascular health. These findings further reinforce the importance of timely intervention, highlighting percutaneous ASD closure as an effective strategy to optimize patient prognosis across different age groups.

Major complications were low across all devices; however, certain device-specific trends in thromboembolic risk were observed. While statistical significance was not achieved, thrombus formation was noted exclusively in the Gore group (3%), consistent with previous findings suggesting a higher incidence of thrombus with Gore devices due to their structural features [20]. In contrast, the absence of device embolization, pericardial effusion, or tamponade in all device groups underscores the general safety of modern ASD occluders. These findings align with the literature, which reports very low incidences of major complications, and reinforce the high safety profile of these devices for percutaneous ASD closure in adults [5,13].

Based on our findings, device selection should be tailored to patient-specific anatomical and clinical factors, as differences in device design influence handling, repositioning, and complication rates. While all occluders provided effective ASD closure and hemodynamic improvements, variations in long-term device stability, thrombus formation, and arrhythmia risk warrant consideration. Clinicians should weigh these factors when selecting an occluder to optimize procedural success and long-term outcomes. Furthermore, structured post-procedural follow-up is crucial to detect potential complications such as residual shunts, device-related thrombus, and embolization. Adequate anticoagulation strategies, particularly in patients with increased thromboembolic risk, should be considered to minimize complications. Our study reinforces the importance of procedural expertise, individualized device selection, and a structured follow-up approach to ensure the best possible patient outcomes.

## 5. Conclusions

Percutaneous ASD closure in adults is safe, effective, and associated with a high procedural success rate, low incidence of residual shunt, and clinically meaningful reductions in pulmonary pressures across different device types. Our study highlights important device-specific variations, particularly the increased risk of atrial fibrillation and thrombus formation associated with the Gore device, underscoring the need for individualized device selection and post-procedural management.

## 6. Limitations

As a retrospective, single-center analysis, the findings may not be fully applicable to other clinical settings or populations. Additionally, the follow-up period of 12 months, while clinically informative, may not fully capture late-onset complications such as device erosion or delayed arrhythmias. Multicenter prospective studies with larger sample sizes and extended follow-up are warranted to confirm these findings and further delineate long-term device-specific outcomes in ASD closure. Furthermore, systematic AF screening during follow-up was not performed since the study was conducted retrospectively. While all patients received a standard resting ECG before discharge, AF was only detected incidentally during routine medical visits or in symptomatic patients who underwent additional ECG or Holter monitoring. This may have led to an underestimation of the post-procedural AF incidence.

## Figures and Tables

**Table 1:** Patients‘ basic characteristics.

	Occlutech ASD Occluder	Amplatzer ASD Occluder	Gore Septal Occluder	All	*p*-Value
	(*n* = 17)	(*n* = 111)	(*n* = 67)	(*n* = 195)	
Age, mean (SD), years	57.1 ± 15.7	52.2 ± 16.5	55.0 ± 15.8	53.6 ± 16.2	0.3
Males, *n* (%)	8 (47)	43 (39)	26 (39)	77 (39.5)	1
BMI, mean (SD), kg/m^2^	25.5 ± 7.3	25.8 ± 5.1	26.2 ± 5.0	24.7 ± 7.5	0.9
Atrial fibrillation/flutter, *n* (%)	4 (23.5)	20 (18.0)	14 (26.4)	38 (19.5)	0.7
Arterial hypertension, *n* (%)	12 (70.6)	49 (44.1)	38 (71.7)	99 (50.8)	0.06
Diabetes mellitus, *n* (%)	0	9 (8.1)	3 (5.7)	12 (6.1)	0.6
Dyslipidemia, *n* (%)	11 (64.7)	30 (27.0)	24 (45.3)	65 (33.3)	<0.01
History of syncope, *n* (%)	1 (5.9)	5 (4.5)	6 (9.0)	12 (6.1)	0.4
Smoking, *n* (%)	4 (23.5)	35 (31.5)	21 (39.6)	60 (30.8)	0.8
Pulmonary hypertension, *n* (%)	5 (29.4)	37 (33.3)	17 (32.1)	59 (30.3)	0.5
PaSys, mean (SD), mmHg	44.4 (18.1)	38.9 (14.6)	36.3 (14.5)	38.4 (14.8)	0.3
Hypermobiles Septum *n* (%)	5 (29.4)	14 (12.6)	11 (16.4)	30 (15.6)	0.2
ASA, *n* (%)	1 (5.9)	19 (17.6)	23 (34.3)	43 (22.4)	<0.01
ASA size, mean (SD), mm	11	14.0 (3.3)	12.0 (1.8)	12.9 (2.8)	0.6
Qp:Qs, *n* (%)	1.5 (0.5)	2.1 (0.9)	1.8 (0.5)	1.8 (0.7)	0.3
Shunt size in % of cardiac output, mean (SD), %	48.0	50.6 (14.3)	43.8 (7.5)	50.0 (13.8)	0.5
Migrane, *n* (%)	2 (11.8)	4 (3.6)	4 (6.0)	10 (5.1)	0.3
Serum creatinine, mean (SD), mg/dl	0.87 ± 0.3	0.85 ± 0.25	0.83 ± 0.3	0.86 ± 0.3	0.7

BMI = body mass index. PaSys = systolic pulmonary pressure. ASA = atrial septal aneurysm. Qp:Qs = ratio between pulmonary (Qp) and systemic flow (Qs).

**Table 2:** Indications for ASD occlusion.

	Occlutech ASD Occluder	Amplatzer ASD Occluder	Gore Septal Occluder	All
	(*n* = 17)	(*n* = 111)	(*n* = 67)	(*n* = 195)
Stroke, *n* (%)	8 (47.1)	16 (14.4)	31 (46.3)	55 (28.2)
TIA, *n* (%)	1 (5.9)	9 (8.1)	3 (4.5)	13 (6.7)
Peripheral embolic event, *n* (%)	1 (5.9)	9 (8.1)	2 (3.0)	12 (6.1)
Hemodynamic relevant shunt, *n* (%)	2 (11.8)	54 (48.6)	14 (20.9)	70 (35.9)
Right heart overload, *n* (%)	4 (23.5)	17 (15.3)	10 (14.9)	31 (15.9)
Primary prevention, *n* (%)	0 (0)	1 (0.9)	3 (4.5)	4 (2.0)
Others, *n* (%)	1 (5.9)	5 (4.5)	4 (6.0)	10 (5.1)

TIA= transient ischemic attack.

**Table 3:** Procedural data.

		Occlutech ASD Occluder	Amplatzer ASD Occluder	Gore Septal Occluder	All	*p*-Value
		(*n* = 17)	(*n* = 111)	(*n* = 67)	(*n* = 195)	
Successful implantation, *n* (%)		17 (100)	100 (90.1)	60 (89.6)	177 (90.8)	0.6
Reasons for unsuccessful implantation, *n*						
	Multifenestrated ASD		1	1		1.0
	Too large ASD		3	1		1.0
	No aortal rim		1	1		1.0
	Too large ASD and no aortal rim		1	2		0.5
	Aortic root riding		1	0		1.0
	High pulmonary pressure		0	1		0.4
	Anatomic variants of heart and vessels		0	1		0.4
	Wrong device size		1	0		1.0
	Other reasons/Unknown		3	0		0.2
Procedure duration, mean (SD), min		48.8 ± 23.6	55.3 ± 23.8	46.5 ± 25.4	51.2 ± 24.6	0.04
Duration of fluoroscopy, mean (SD), min		8.4 ± 5.3	9.7 ± 6.7	7.0 ± 6.0	8.5 ± 6.4	0.02
largest occlude disc, mean (SD), mm		22.7 ± 8.5	20.7 ± 5.7	30.6 ± 4.9		

**Table 4:** Post-procedural events during the first 12 months.

		Occlutech ASD Occluder	Amplatzer ASD Occluder	Gore Septal Occluder	All	*p*-Value
WHO classification of bleeding						
	Minor (grades 0–2), *n* (%)	0	0	0	0	NA
	Major (grades 3 and 4), *n* (%)	0	0	0	0	NA
Vascular access site complications, *n* (%)		0	1 (0.9)	1 (1.5)	2 (1.0)	1
Pericardial tamponade, *n* (%)		0	0	0	0	NA
Device embolism or dislocation, *n* (%)		1 (5.9)	0	1 (1,5)	2 (1.0)	0.07
Erosions, *n* (%)		0	0	0	0	NA
New onset of atrial fibrillation, *n* (%)		0	1 (0.9)	5 (7.5)	6 (3.1)	0.05
New onset of atrial flutter, *n* (%)		0	0	0	0	NA
TIA, *n* (%)		0	0	0	0	NA
Stroke, *n* (%)		0	0	2 (3.0)	2 (1.0)	0.3
Device thrombus, *n* (%)		0	0	2 (3.0)	2 (1.0)	0.3
Pulmonary embolism, *n* (%)		0	0	0	0	NA
Death, *n* (%)		0	0	1 (1,5)	1 (0.5)	0.4

TIA = transient ischemic attack. NA = Not available.

## Data Availability

The original contributions presented in the study are included in the article.

## Supplementary Materials

The following supporting information can be downloaded at: https://www.mdpi.com/article/10.3390/jcm14061867/s1, Table S1: Periprocedural complications.
