# Supplementary material for: Efficacy and Safety of Percutaneous ASD Closure in Adults: Comparative Outcomes of Occluder Devices in a Single-Center Cohort
Source: J Clin Med. 2025 Mar 10;14(6):1867. doi: 10.3390/jcm14061867 (PMC11942770; doi:10.3390/jcm14061867)
Supplement: Supplementary file 1 [file jcm-14-01867-s001.zip › jcm-3464225-supplementary.pdf]

Supplementalry Table S1: Periprocedural complications

	Occlutech ASD Occluder	Amplatzer ASD Occluder	Gore Septal Occluder	All	p-value
**Hemodynamically significant pericardial effusion, n (%)**	0	0	0	0	NA
**New onset of atrial fibrillation, n (%)**	0	0	0	0	NA
**TIA, n (%)**	0	0	0	0	NA
**Stroke, n (%)**	0	0	0	0	NA
**Device dislocation, n (%)**	0	1 (0.9)	0	1 (0.5)	1
**Puncture site complications, n (%)**	0	0	1 (1.6)	1 (0.5)	0.4
**WHO classification of bleeding**					
Minor (grades 0-2), n (%)	0	0	0	0	NA
Major (grades 3 and 4), n (%)	0	0	0	0	NA
**Death, n (%)**	0	0	0	0	NA

TIA = Transient ischemic attack. NA = not applicable
